# Supplementary material for: Magnesium ions improve vasomotor function in exhausted rats
Source: PLoS One. 2023 Feb 13;18(2):e0279318. doi: 10.1371/journal.pone.0279318 (PMC9925009; doi:10.1371/journal.pone.0279318)
Supplement: S1 Dataset — (DOCX) [file pone.0279318.s001.docx]

Figure1


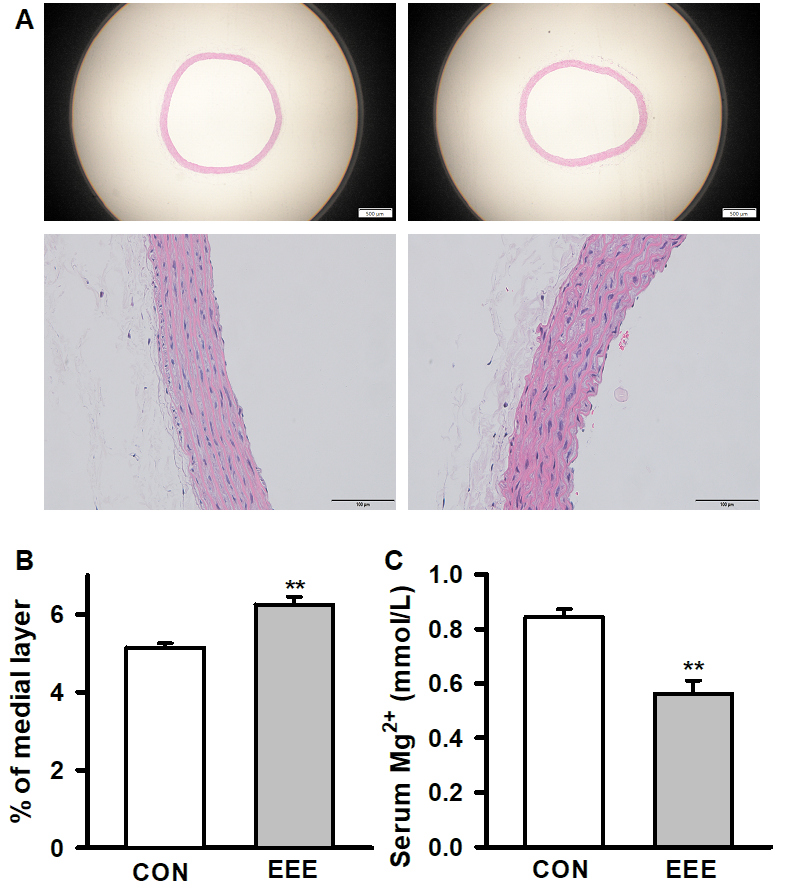


Fig.1 Effects of exhaustive exercise on morphology of thoracic aorta and serum Mg^2+^ in SD rats. (A) left pictures are representative images of CON group taken at 40× magnification and 200× magnification, respectively and right pictures are representative images of EEE group taken at 40× magnification and 200× magnification, respectively. (B) The ratio of thoracic aortic thickness in CON and EEE groups. (C) The concentration of serum Mg^2+^ in each group. Data presented as mean ± SE. **P* < 0.05 and ***P* < 0.01 compared with the CON group.

(B) The ratio of thoracic aortic thickness:

CON: 4.7869 4.4813 4.9392 4.7726 4.1861 4.6087 5.4259 5.4703 5.6918 5.5719 5.5875 5.5711 5.3651 4.8531 5.493 5.1414 5.5416 5.1779

(n=18)

Mean: 5.1481 SD: 0.4481 SE: 0.1056

EEE: 5.9219 7.0302 6.3767 6.2689 7.18 6.994 5.6469 6.7268 5.0598 5.7376 6.6877 5.3726 (n=12)

Mean: 6.2502 SD: 0.6995 SE: 0.2019

(C) The concentration of serum Mg^2+^ in each group:

CON: 0.771 0.7809 0.7759 0.8204 0.9835 0.7759 0.7809 0.8797 0.7661 1.0428 0.9044 (n=11)

Mean: 0.8438 SD: 0.0965 SE: 0.0291

EEE: 0.3032 0.5042 0.7152 0.6851 0.7605 0.7203 0.4137 0.5595 0.4791 0.4841 (n=10)

Mean: 0.5625 SD: 0.1521 SE: 0.0481

Figure2


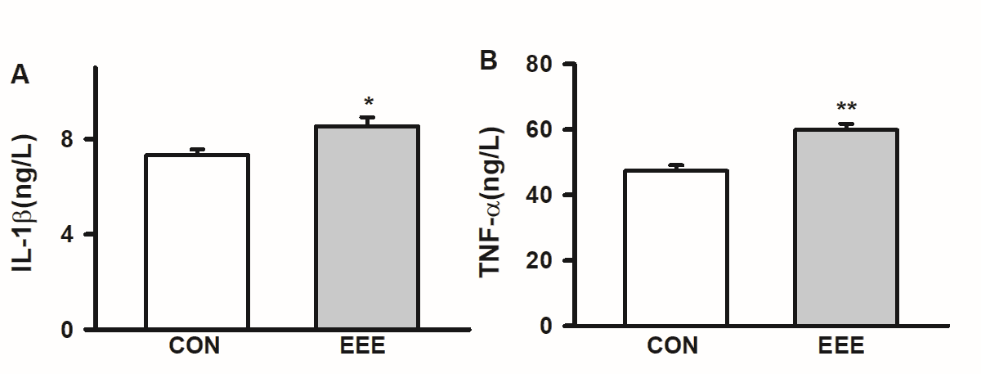


Fig.2 Effects of exhaustive exercise on serum Il-1β and TNF-α in SD rats. The concentration of IL-1β and TNF-α detected in serum from two groups. Data presented as mean ± SE. **P* < 0.05 and ***P* < 0.01 compared with the CON group.

(A) The concentration of IL-1βdetected in serum from two groups.

CON: 6.5876 7.9452 7.7683 7.3433 6.6059 7.6856 (n=6)

Mean: 7.3227 SD: 0.5954 SE: 0.2431

EEE: 7.6328 7.4031 9.7669 9.7439 8.0555 8.1956 9.0042 (n=7)

Mean: 8.5431 SD: 0.9695 SE: 0.3664

(B) The concentration of TNF-α detected in serum from two groups.

CON: 47.8332 45.3466 55.5091 52.7342 48.6981 42.3375 45.0763 41.4186 (n=8)

Mean: 47.3692 SD: 4.8858 SE: 1.7274

EEE: 62.6805 59.9416 55.5271 52.6441 59.3831 58.4641 53.473 70.3023 68.9869 57.2028 (n=10)

Mean: 59.8606 SD: 5.9738 SE: 1.8891

Figure3


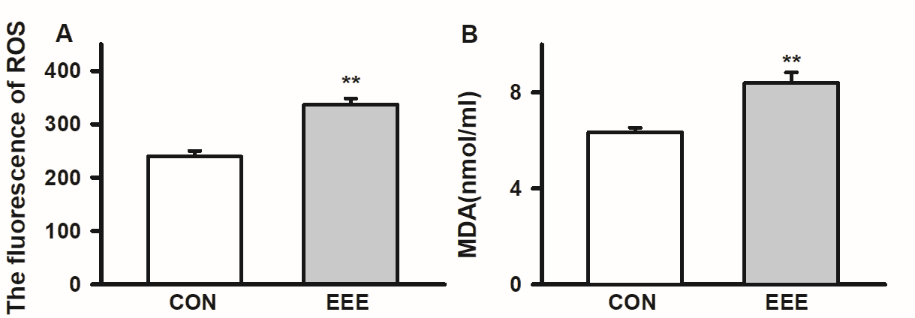


Fig.3 Effects of exhaustive exercise on serum ROS and MDA in SD rats. The concentration of ROS and MDA detected in serum from two groups. Data presented as mean ± SE. **P* < 0.05 and ***P* < 0.01 compared with the CON group.

(A) The fluorescence of ROS detected in serum from two groups

CON: 248.5 215.9 206.6 275 201.3 228.3 283.8 254.7(n=8)

Mean: 239.2625 SD: 31.0722 SE: 10.9857

EEE: 383.3 350.3 299.7 363.6 339.6 296.6 284.4 362.8 377.5 307.6 (n=10)

Mean: 336.54 SD: 36.5046 SE: 11.5438

(B) The concentration MDA detected in serum from two groups

CON: 6.858 6.765 6.321 6.379 5.324 6.388 5.821 6.858(n=8)

Mean: 6.3392 SD: 0.5376 SE: 0.1901

EEE: 6.94 6.954 9.878 9.968 9.125 8.28 6.306 9.907 9.056 7.547(n=10)

Mean: 8.3961 SD: 1.3832 SE: 0.4374

Figure4


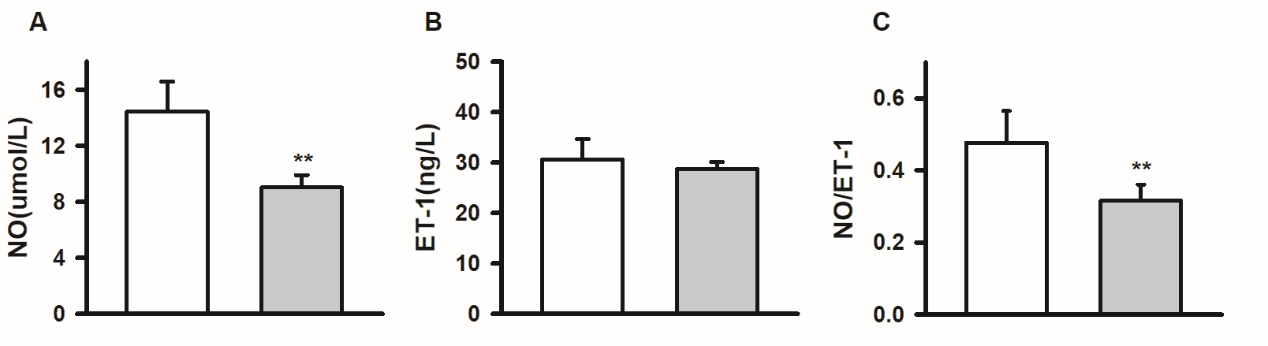


Fig.4 Effects of exhaustive exercise on serum NO and ET-1 in SD rats. (A) and (B)The concentration of NO and ET-1 detected in serum from two groups. (C) The results of the ratio of NO/ET-1 in the two groups. Data presented as mean ± SE. **P* < 0.05 and ***P* < 0.01 compared with the CON group.

(A) The concentration of NO detected in serum from two groups.

CON: 16.327 14.622 10.901 15.643 11.322 16.227 15.906 14.469(n=8)

Mean: 14.4271 SD: 2.159 SE: 0.7633

EEE: 10.407 8.701 9.175 8.001 8.791(n=5)

Mean: 9.015 SD: 0.886 SE: 0.3962

(B) The concentration of ET-1 detected in serum from two groups.

CON: 27.8505 33.7528 27.6382 28.3282 29.6764 38.636 26.5661 32.1711(n=8)

Mean: 30.5774 SD: 4.0638 SE: 1.4368

EEE: 26.789 28.1478 28.6679 29.7507 30.2815(n=5)

Mean: 28.7274 SD: 1.3747 SE: 0.6148

(C) The ratio of NO/ET-1 in the two groups

CON: 0.5862 0.4332 0.3944 0.5522 0.3815 0.42 0.5987 0.4498(n=8)

Mean: 0.477 SD: 0.088 SE: 0.0311

EEE: 0.3885 0.3091 0.32 0.2689 0.2903(n=5)

Mean: 0.3154 SD: 0.0453 SE: 0.0202

Figure5


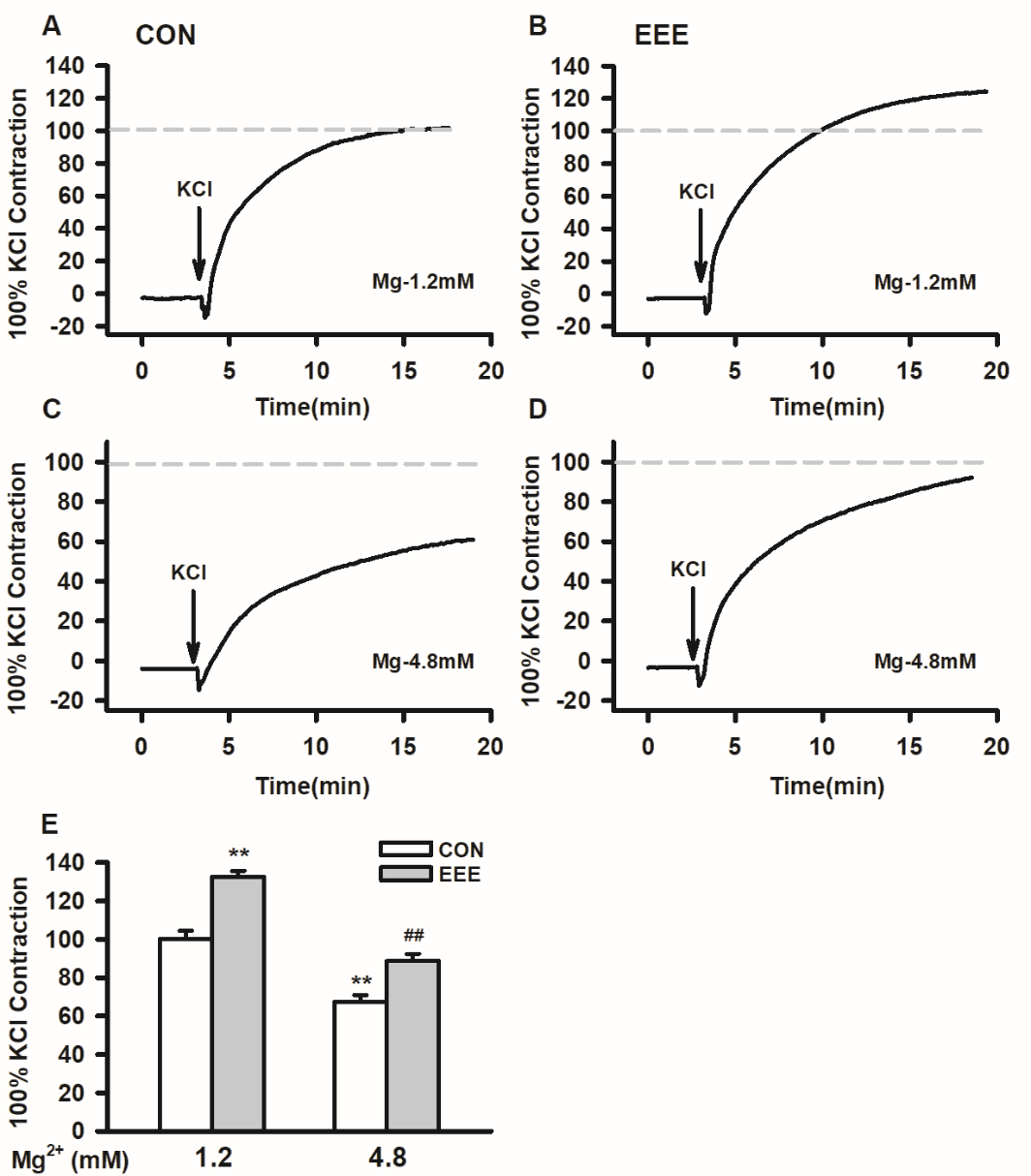


Fig.5 The effect of magnesium on the KCl-induced contraction response in thoracic aortic from two group rats. (A–D) Typical traces showing KCl -induced contraction responses in thoracic aortic at 1.2 and 4.8 mM magnesium, respectively. (E) Bar graphs showing the average values of the maximal contraction. Data are expressed as percentages of the 60 mM K^+^-induced contractile response in con treated by 1.2mM magnesium. ***P <* 0.01 or ^##^*P <* 0.01 compared with 1.2 mM magnesium in each group. Data are presented as means ± SE.

(E) The average values of the maximal contraction

1.2mM Mg^2+^-CON (%): 76 110 80 66 73 53 77 68 91 92 73 102 100 99 121 95 91 102 108 91 107 94 89 84 101 70 145 116 139 106 150 154 150 128 (n=34)

Mean: 100.0294 SD: 25.9364 SE: 4.4481

1.2mM Mg^2+^-EEE (%): 99 102 143 121 125 131 123 130 114 165 147 149 119 156 135 154 132 159 152 172 158 116 121 115 125 141 113 141 102 153 117 125 113 138 118 147 134(n=37)

Mean: 132.5676 SD: 18.7076 SE: 3.0755

4.8mM Mg^2+^-CON (%): 63 51 55 43 60 55 73 76 59 59 57 53 96 62 67 75 106 74 92 71(n=20)

Mean: 67.35 SD: 16.0009 SE: 3.5779

4.8mM Mg^2+^-EEE (%): 110 73 65 70 89 85 76 125 102 104 77 86 49 67 62 108 82 89 68 137 99 103 88 110 83 93 92 (n=27)

Mean: 88.5926 SD: 20.0236 SE: 3.8535

Figure6


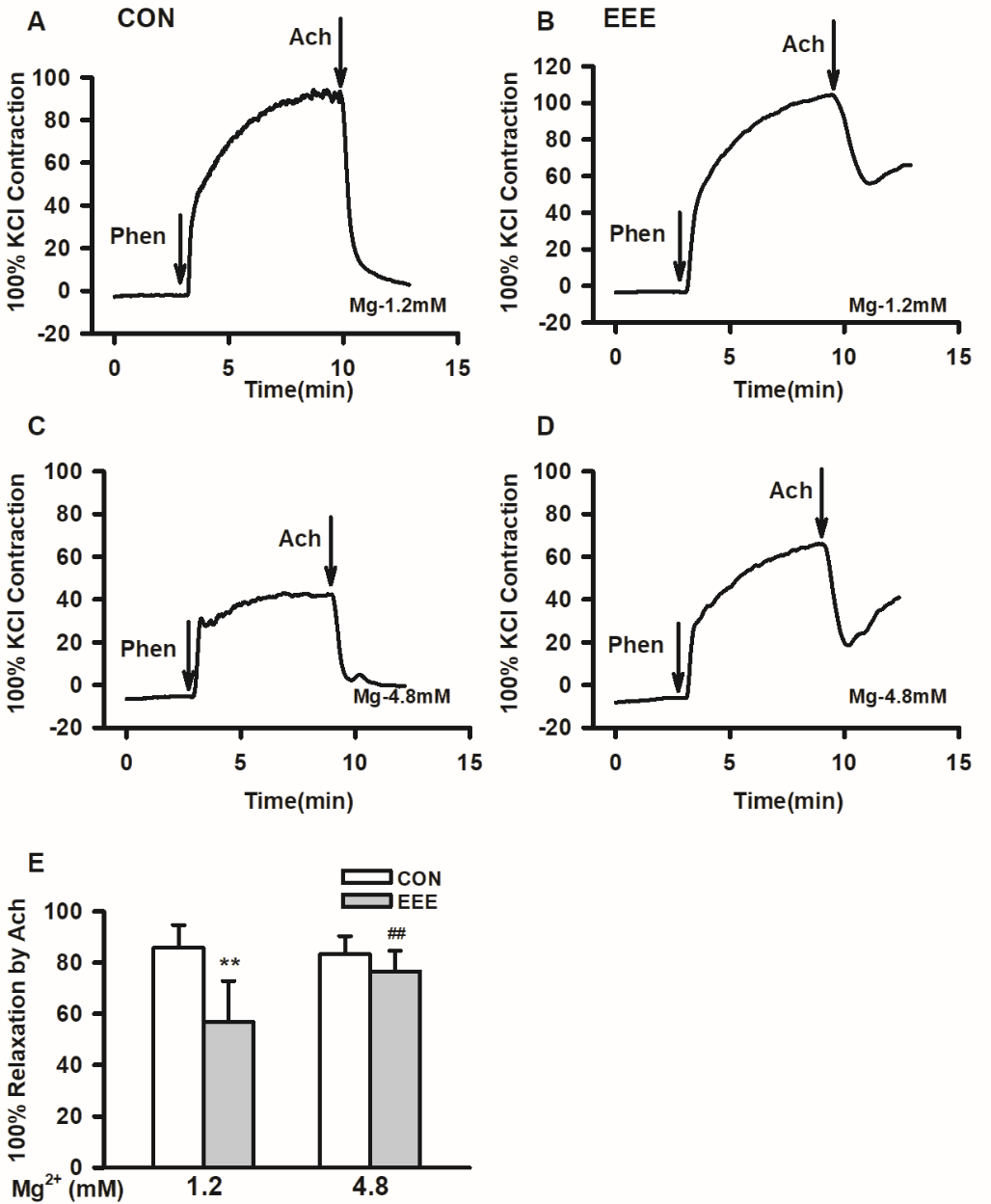


Fig.6 The effect of magnesium on the acetylcholine (ACh)-induced relaxant response in thoracic aortic from two group rats. (A–D) Typical traces showing ACh-induced relaxant responses in thoracic aortic at 1.2 and 4.8 mM magnesium, respectively. (E) Bar graphs showing the average values of the maximal Relaxation. Abbreviation: Phen, phenylephrine. ***P <* 0.01 or ^##^*P <* 0.01 compared with 1.2 mM magnesium in each group. Data are presented as means ± SE.

(E) The average values of the maximal Relaxation

1.2mM Mg^2+^-CON (%): 90.9292 80.0621 91.9869 87.9509 91.7141 77.2472 84.2444 68.9802 90.4686 89.4483 93.0295 94.5885 90.884 92.0143 95.2663 93.0283 80.0241 90.885 81.5461 94.2511 75.288 77.9964 62.5134 (n=23)

Mean: 85.8412 SD: 8.8648 SE: 1.8484

1.2mM Mg^2+^-EEE (%): 63.0901 58.8919 39.2956 59.8146 67.4123 44.2438 62.1089 50.7822 38.6461 77.9893 74.4943 71.6398 36.2445 78.1421 29.9521 71.9971 77.5564 60.789 62.268 26.9869 64.4554 38.3552 53.2639 (n=23)

Mean: 56.8878 SD: 15.9721 SE: 3.3304

4.8mM Mg^2+^-CON (%):83.8318 89.033 75.039 79.1425 94.7735 92.4084 92.8696 90.1961 74.7157 79.9242 76.3636 77.9817 90.5983 85.8099 85.9877 88.024 71.1548 79.6296 76.0907 83.7013 (n=20)

Mean: 83.3638 SD: 7.0189 SE: 1.5695

4.8mM Mg^2+^-EEE (%):72.3315 62.99 82.2699 90.2945 83.4793 72.2946 82.9801 80.9796 75.3636 76.8496 77.6718 61.519 83.7518 72.5297 84.5411 65.2439 81.1927 73.8158 (n=18)

Mean: 76.6721 SD: 7.9245 SE: 1.8678
